# Comparison of different plaque indices with regard to sensitivity and specificity for the quantification of plaque during orthodontic therapy

**DOI:** 10.1038/s41598-022-24817-y

**Published:** 2022-12-05

**Authors:** Christina Erbe, Teresa Temming, Daniela Ohlendorf, Irene Schmidtmann, Ambili Mundethu, Priscila Ferrari-Peron, Heinrich Wehrbein

**Affiliations:** 1 Department of Orthodontics, University Medical Center of the Johannes Gutenberg-University, Augustusplatz 2, 55131 Mainz, Germany; 2 Institute of Occupational, Social and Environmental Medicine, Goethe-Universiät, Frankfurt, Germany; 3 Institute for Medical Biostatistics, Epidemiology and Informatics (IMBEI), University Medical Centre of the Johannes Gutenberg-University, Mainz, Germany

**Keywords:** Dental education, Dental public health, Orthodontics

## Abstract

To compare four plaque indices used in orthodontics. An objective, quantitative plaque index and three subjective conventional plaque indices were analyzed. The study included n = 50 photographs of n = 50 subjects with a multibracket appliance (MB) in the maxilla and mandible. Photographs were taken using *Digital Plaque Imaging Analysis* (DPIA) and the *Percentage Plaque Index* (PPI) was calculated. The conventional plaque indices, a modified version of the Turesky-modification of the Quigley & Hein Index (TQH index), Attin index, and modified *bonded bracket index* (mBB index) were collected from n = 14 evaluators using the DPIA photographs. The evaluators had different levels of orthodontic experience: n = 4 evaluators had little orthodontic experience, n = 5 evaluators had moderate orthodontic experience, and n = 5 evaluators had a lot of orthodontic experience. Plaque accumulation was assessed differently with the plaque indices. Thus, the plaque indices are not interchangeable. We recommend DPIA as an objective, quantitative and sensitive method for plaque determination in scientific studies. The simple statistical evaluation offers a great advantage over conventional plaque indices.

## Introduction

Oral hygiene plays a prominent role in dental health. Caries is one of the most common non-communicable diseases in modern society^1^. For orthodontic patients whose treatment extends over a longer period of time, oral hygiene is of particular importance. Patients treated with multibracket appliances (MB) show increased plaque accumulation and increased gingivitis^2–4^. The physiological cleaning mechanism of oral muscles and saliva is restricted by the irregular surface of the MB^5^. In addition, studies have shown altered microbial flora in patients with MB^4,6–9^.

Plaque indices are applied in order to determine the amount of plaque present. On the one hand, plaque indices are an important method for investigating the efficiency of oral hygiene products in scientific studies^10–14^. On the other hand, plaque indices promote patients’ motivation to improve oral hygiene in daily practice^15^. Patients with MB have different predilection sites than patients without MB. At this time, few studies are available that have investigated the use of dental plaque indices in patients with MB^16^. There is little data available for the use of orthodontic plaque indices regarding reliability and reproducibility in clinical studies^16^. Furthermore, dental plaque indices have rarely been compared with orthodontic plaque indices^16^. Subjective, conventional plaque indices can be distinguished from objective, quantitative plaque determination methods such as *Digital Plaque Imaging Analysis* (DPIA)^2^. However, subjective plaque indices represent the majority of methods used for the evaluation of oral hygiene^2,17^. So far, the DPIA method has only been used in a few cases in orthodontic patients with MB^2,14^.

The purpose of our study was to evaluate the agreement of established subjective plaque indices with an objective computerized plaque quantification via the DPIA under almost real-world conditions, comparing the *Percentage Plaque Index* (PPI)—an objective plaque determination method with the conventional plaque indices—a subjective plaque determination method^18^. Among the conventional plaque indices, a modified version of the Turesky-modification of the Quigley & Hein Index (TQH index), Attin index, and modified *Bonded Bracket index* (mBB index) were investigated. Secondary objective is to compare different groups of examiners (experienced vs unexperienced) with respect to agreement of established subjective plaque indices with an objective computerized plaque quantification via the DPIA.

## Materials and methods

### Survey of the four plaque indices

**Figure 1:**
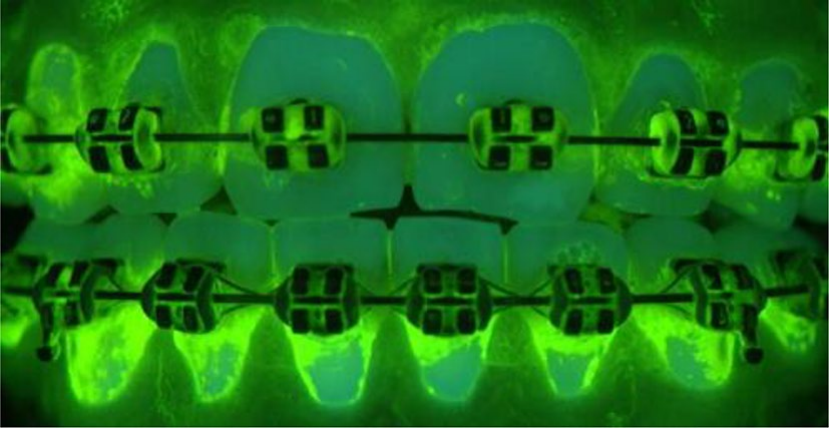
DPIA photo of a subject.

In the present study, n = 50 photographs were used, which were created and calculated according to the DPIA introduced by Klukowska et al.^2^. The DPIA photographs were based on n = 50 subjects. One photograph was used per subject. The procedure allowed the determination of the PPI to determine the percentage of plaque accumulation on the tooth surface^2^. Figure 1 shows an example of a DPIA photo of a subject. Similar to the previously described imaging system by White et al. and White^19,20^ the imaging system used in this study consisted of 2 long-wave UV flash units (model FX60, Balcar Company, Strasburg, France) and a powerpack (Starflash 2, Balcar) equipped with filters to remove most of the visible light. Plaque was made visible with a fluorescein rinsing solution, as previously described by Klukowska et al.^2^.

All images were acquired under standardized room conditions, with all other light sources excluded, and automatically saved on the computer, managing the image database and a log of acquisition time, date, operator, patient, visit number, and standardization checks. The plaque assessment is quantitatively driven by the established computer decision rule. Since no subjective assessment by the clinician is required, the DPIA, in this context, is operator-independent, and no subjective clinical effects are allowed or expected^20^.

The n = 50 subjects had a mean age of 13.9 ± 1.98 years and were previously examined at the Department of Orthodontics, University Medical Center of the Johannes Gutenberg-University, Mainz (Rhineland-Palatinate, Germany). All subjects had an MB in situ in the maxilla and mandible at the time of the study. The study was approved by the Freiburg Ethics Committee International (Baden-Württemberg, Germany) (feki code 07/2113). Details of the study design can be obtained from the Freiburg Ethics Committee.

DPIA photographs were also used to obtain conventional plaque indices by n = 14. The vestibular tooth surfaces of all twelve anterior teeth (six maxillary, six mandibular) were examined. The raters had varying levels of orthodontic experience. At the time of the study, n = 10 students during postgraduate orthodontic training and n = 4 raters were orthodontic specialists at the Department of Orthodontics, University Medical Center, Johannes Gutenberg University, Mainz, Germany. n = 4 raters had little (≤ 1 year), n = 4 raters had moderate (≥ 2 years to ≤ 5 years), and n = 5 raters had a lot (> 5 years) of orthodontic experience. Each evaluator assessed plaque accumulation for all subjects and collected each of the three conventional plaque indices once. All evaluators had gained a lot of experience with all the plaque indices investigated during their undergraduate as well as postgraduate training and during daily work in practice. Before participation in this study, they received detailed instruction and written description of the plaque indices complemented by a pictorial explanation for the evaluation. The following conventional plaque indices were examined:

The TQH index represents a modification of the Turesky index according to Cugini et al.^21,22^. According to the modification, the vestibular tooth surface was divided into three regions (mesial, central, distal). The Turesky index is an international standard plaque index^23^. Plaque accumulation was classified into the following assessment grades^22^: grade 0—no plaque, grade 1—single plaque islands along the gingival margin, grade 2—thin plaque line (≤ 1 mm) along the gingival margin, grade 3—plaque line > 1 mm, plaque covers ≤ 1/3 of the tooth surface, grade 4—plaque covers ≤ 2/3 of the tooth surface, grade 5—plaque covers > 2/3 of the tooth surface.

As an orthodontic plaque index, the Attin index is based on plaque formation in patients with MB^24^. Plaque accumulation was classified into the following assessment grades: grade 0—no visible plaque, grade 1—plaque islands on the proximal surfaces, grade 2—plaque islands cervical to the bracket in addition to the proximal surfaces, grade 3—plaque covers > 1/3 of the surface cervical to the bracket.

The mBB index according to Delaurenti et al.^25^ consists of a combination of a dental and an orthodontic plaque index. The scoring levels are based on those of the Oral Hygiene Index according to Greene and Vermillion^26^. The classification of vestibular tooth areas was based on Williams et al.^27^. Each tooth was divided into four areas (incisal, distal, mesial, gingival). Plaque accumulation was classified into the following assessment grades: grade 0—no plaque, grade 1—plaque covered ≤ 1/3 of the tooth surface, grade 2—plaque covered ≤ 2/3 of the tooth surface, grade 3—plaque covered > 2/3 of the tooth surface.

### Statistical analysis

In this study, the results of all four plaque indices were processed in Excel 2013 (Microsoft Office 2013, Microsoft Corporation, Redmond, WA, USA). Statistical analysis was performed using SAS 9.4 statistical software (SAS Institute, Cary, NC, USA) and R 3.2.3 (The R Foundation for Statistical Computing, 2016, download: https://cloud.r-project.org). To achieve comparability the plaque values of the TQH, Attin, and mBB indices with the plaque values of the PPI, all plaque values of the conventional plaque indices were converted to percentage plaque values as follows:

$$\text{Plaque index (\%)=}\frac{\frac{\text{Total of plaque values}}{\text{Number of surfaces}}}{\text{Highest graduation score}} \, \times {100}.$$

Thus, all indices are standardized and should be exchangeable. In this study, only the percentage plaque values were used.

The agreement of two plaque indices related to the classification of the subjects into the same oral hygiene category was investigated using Cohen’s kappa^28^. For this purpose, the percentage plaque values of the four plaque indices were classified into the oral hygiene categories according to Lange^29^. The agreement of two plaque indices related to the percentage plaque values was evaluated with the Bland–Altman plots^30,31^. The study with the Bland–Altman plots was considered without and with the orthodontic experience of the evaluators. In Bland–Altman plots, differences between two measurements are plotted against the mean of the two measurements. Three horizontal lines are shown: the mean difference of the measurements (bias) and the lower and upper limits of agreement. 95% of the differences lie between lower and upper limits of agreement. The statistical analysis with the Bland–Altman diagrams allows a comparison of the clinical tolerance range with the *Limits of Agreement* (LoA) and thus an evaluation of the comparability for clinical use. The clinical tolerance range was set at ± 10%. i.e. if the upper limit is greater than 10% or the lower limit of agreement is less than − 10%, there is no clinically acceptable difference.

### Ethics approval and consent to participate

All investigations and procedures were conducted according to the principles expressed in the Declaration of Helsinki.

### Approval for human experiments

The study was approved by an Institutional Review Board (No. 07/2113). Informed consent was obtained from each subject and their legal guardians for the digital photographs used in this study.

## Results

### Comparison of plaque indices related to the classification of subjects in the same oral hygiene category

**Figure 2:**
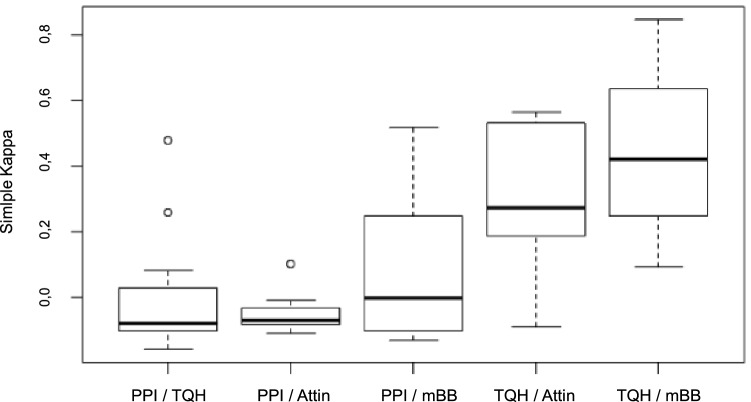
Summary representation of simple kappa in a boxplot diagram. The comparisons of the four plaque indices (TQH-, Attin-, mBB-index, PPI) related to the classification of the subjects into the same oral hygiene category are shown. For each rater, the simple kappa was determined for the comparison of two plaque indices. The individual box plots represent the distribution of simple kappas across raters with different pairs of plaque indices.

In the comparisons of the conventional plaque indices with each other, a higher simple kappa was achieved than in the comparisons of the PPI with the conventional plaque indices (Fig. 2). Thus, the agreement among the conventional plaque indices was significantly better than the agreement of the PPI with the conventional plaque indices. The most inadequate agreement was obtained when comparing the PPI with the TQH index (simple kappa − 0.01 [CI − 0.06; 0.03]), or Attin index (simple kappa − 0.05 [CI − 0.08; − 0.03]), while the comparison between the TQH and mBB indexes (simple kappa 0.44 [CI 0.38; 0.49]) obtained the best agreement.

The oral hygiene of the subjects was frequently classified in a higher oral hygiene category with the conventional plaque indices than with the PPI (Table 1). When the conventional plaque indices were compared with each other, the oral hygiene of the subjects tended to be classified in a higher oral hygiene category with the Attin index than with the TQH index. The mBB index tended to place oral hygiene in a lower oral hygiene category than the TQH index.

**Table 1:** Cross-tabulation showing concordance and discordance in percent for comparison of PPI with TQH index related to classification of subjects into oral hygiene categories.

PPI	TQH Index			
	Very good	Good	Moderate	Insufficient
Very good	**0.6**	1.3	2.1	0.00
Good	0.1	**2.3**	25.0	2.6
Moderate	0.4	1.9	**19.7**	34.0
Insufficient	0.00	0.00	0.6	**9.4**

Significant values are in bold.

### Comparison of the plaque indices in relation to the percentage plaque values

**Figure 3:**
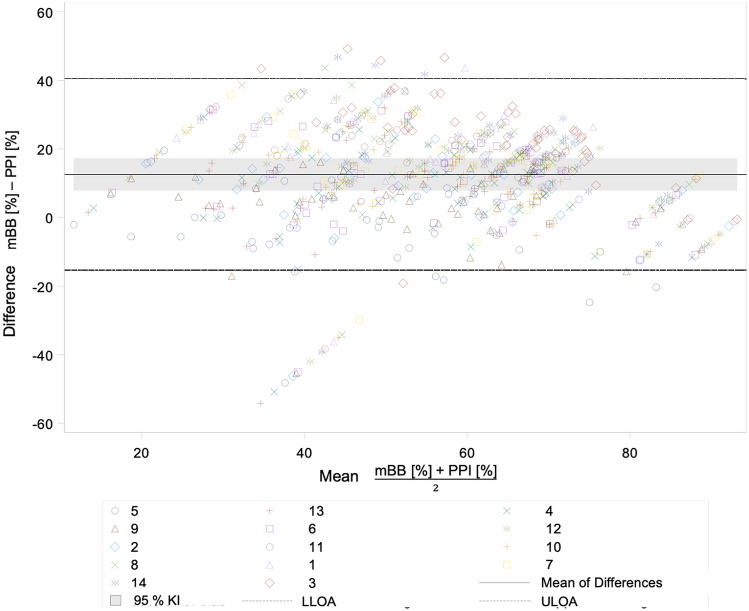
The Bland–Altman plot for the comparison of the plaque values of the PPI with the plaque values of the mBB index.

Comparison of the PPI with the Attin index revealed the largest systematic error for the Bland–Altman plot, with a *bias* of 31.6 [95% CI 26.7; 36.5]. Comparison of the PPI with the TQH index yielded a larger systematic error with a *bias* of 15.7 [95% CI 10.8; 20.7] than comparison of the PPI with the mBB index with a *bias* of 12.5 [95% CI 7.8; 17.3]. Consequently, the PPI and the mBB index showed the smallest systematic error among the comparisons of the PPI with the conventional plaque indices (Fig. 3).

**Figure 4:**
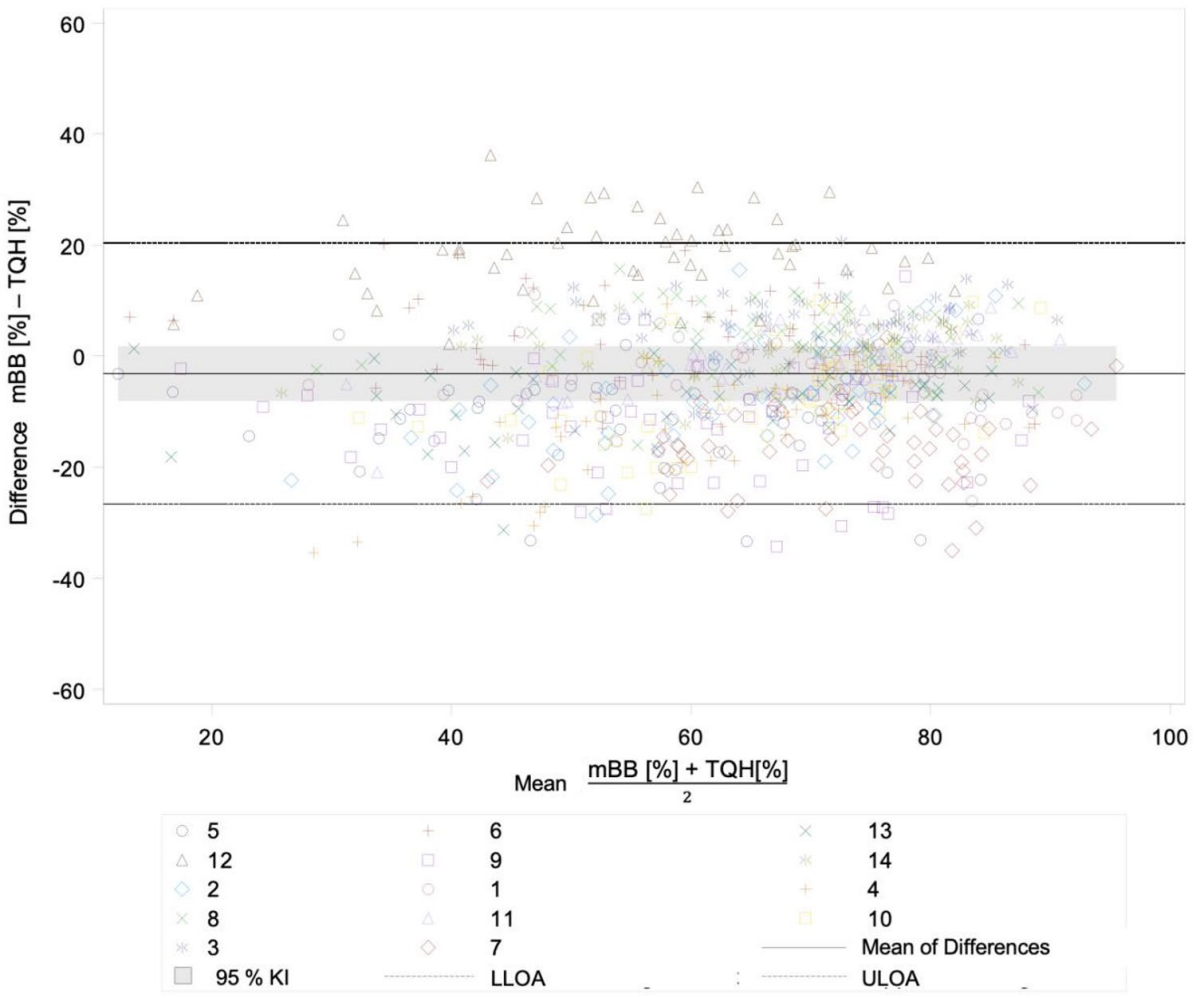
The Bland–Altman plot comparing plaque values of TQH index with plaque values of mBB index.

Among the conventional plaque indices, the TQH and mBB indices yielded the best matching plaque values (Fig. 4). Both plaque indices had the lowest systematic error with a *bias* of − 3.2 [95% CI − 8.1; 1.8] compared to the other comparisons. On average, the mBB index measured only slightly lower plaque values than the TQH index. The Attin and TQH indices agreed less, with a *bias* of 15.9 [95% CI 12.3; 19.4]. On average, the Attin index yielded higher plaque values than the TQH index.

The greatest dispersion occurred when comparing the PPI with the Attin index, with a width of LoA of 65.6 [LLoA − 1.2; UloA 64.4]. LoA were similar when comparing the PPI with the TQH index [LLoA OA − 11.9; ULoA 43.4] and when comparing the PPI with the mBB index [LLoA − 5.3; ULoA 40.4]. Thus, in this study, there was an insufficient correlation between the PPI, the objective plaque determination method, and the TQH index, a modification of the international standard plaque index. Comparison among conventional plaque indices yielded slightly better results. LoA were similar when comparing the TQH index with the Attin index [LLoA − 7.2; ULoA 39.0] and when comparing the TQH index with the mBB index [LLoA − 26.7; ULoA 20.4].

**Figure 5:**
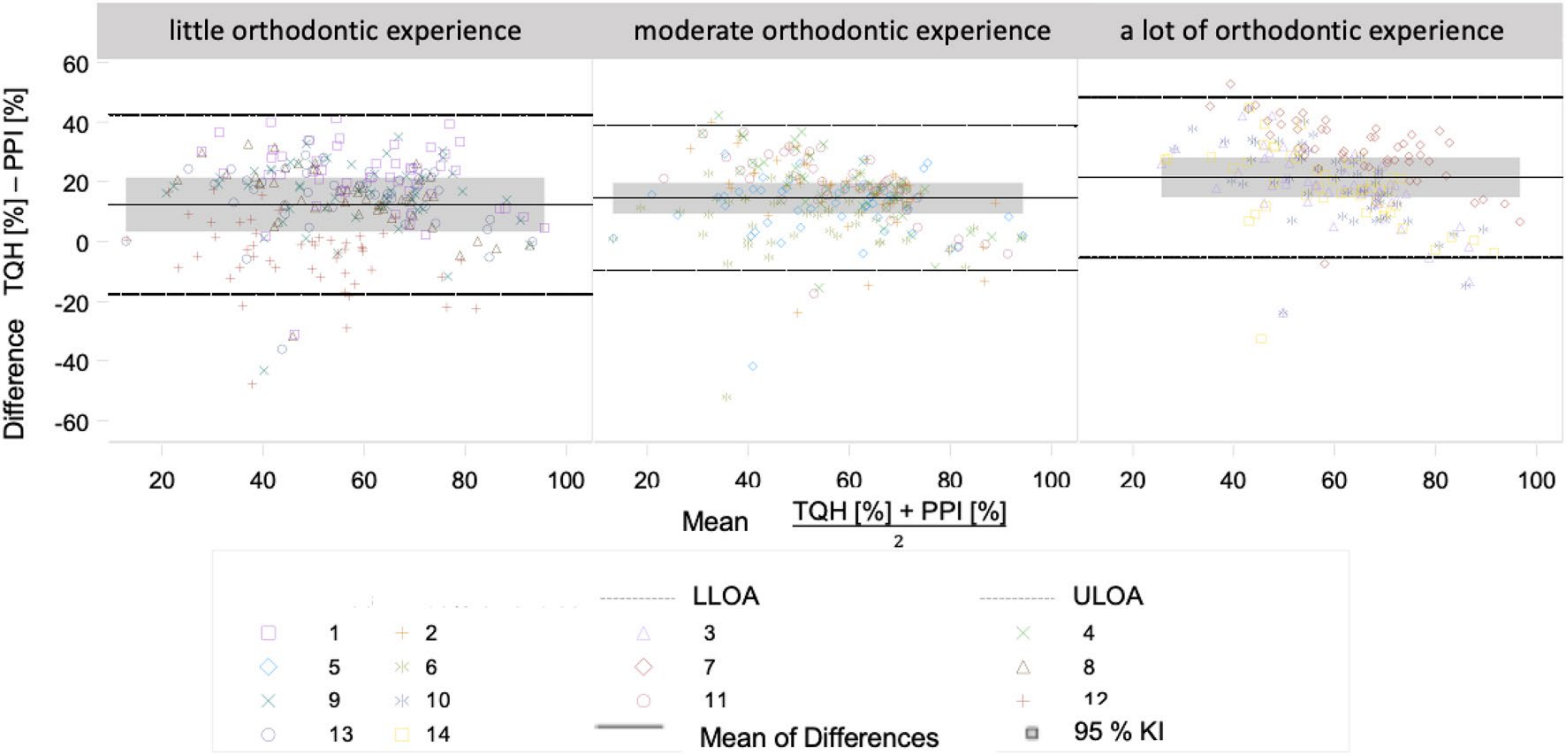
Bland–Altman diagram for the comparison of the plaque values of the PPI with the plaque values of the TQH index with consideration of the orthodontic experience of the evaluators.

The orthodontic experience of the evaluators only slightly influenced the results of the Bland–Altman diagrams. On average, the evaluators with a lot of orthodontic experience showed a larger *bias* than the evaluators with little or moderate orthodontic experience (Fig. 5). On average, the evaluators with little orthodontic experience had slightly larger LoA than the evaluators with moderate or a lot of orthodontic experience. Thus, the scatter in the comparisons of two plaque indices was larger for the evaluators with little orthodontic experience than for the other evaluator groups.

## Discussion

Conventional plaque indices have been used for some time in studies to assess plaque accumulation and are considered the standard method^2,17^. The advantages of conventional plaque indices are their practical, simple application and low instrumental and time requirements^32^. However, according to Sagel et al.^33^ and Heintze et al.^3^ the application of subjective as well as conventional plaque indices provide non-specific results and therefore complicate diagnoses which results in higher costs and a more complex study design. In addition, special attention must be paid to the statistical evaluation of conventional plaque indices^17,34–36^.

Therefore, in scientific studies, DPIA is superior to conventional plaque indices as an objective, true quantitative method for plaque determination in vivo^2,14,19,20,33^, achieves good reproducibility^2,33^ and is reported to be more sensitive and precise compared with conventional plaque indices^14^, even though only anterior teeth have been evaluated using the DPIA method^2,14,37–40^ which is a limit of the method. Nevertheless, if a conventional plaque index is used in studies, the use of an orthodontic plaque index or a dental plaque index modified for patients with MBA is recommended. Al-Anezi and Harradine^41^ cited the complexity of the DPIA method as a disadvantage. The digital storage of the photographs and consequently their use for longitudinal studies is an overall advantage^42^.

In the literature, conventional plaque indices have rarely been compared with digital photo-analysis methods such as DPIA for quantitative plaque determination. In 1993, Soder et al.^43^ compared the results of the PPI with the Turesky index in a study of computerized planimetric plaque measurement. For each degree of the Turesky index, the PPI yielded a wide range of plaque values. The variable range of the PPI increased as the Turesky index was graduated. Due to the structure of the scoring grades of the Turesky index, which assessed small plaque accumulations more accurately than larger plaque accumulations. The study by Han et al.^44^ showed a moderate correlation between the PPI and the Turesky index or the Silness and Löe index. However, a digital photo analysis method other than DPIA was used. In addition, a different statistical analysis was used to compare the PPI with the Turesky index than in our study. Furthermore, the Turesky index was collected from only n = 2 raters. In contrast to the present study, Rosa and Elizondo^45^ determined a strong correlation between the PPI and the Turesky index. However, as in Han et al. a different statistical analysis was used, and only n = 1 rater performed the collection of the Turesky index. In addition, the equipment used to perform the digital photo analysis was significantly different from the equipment used in the DPIA procedure as applied in our study. Carter et al.^42^ compared the results of the DPIA method with the *modified Navy Plaque Index* according to Rustogi et al.^46^. When the two plaque determination methods were compared, there was a relatively low correlation. Carter et al.^36^ concluded that the conventional plaque index did not quantitatively assess plaque accumulation. Instead, Carter et al.^42^ stated that the conventional plaque index assessed spatial plaque spread on the tooth surface regardless of quantity.

In this study, the greatest agreement among the 14 assessors was obtained with the Attin index [ICC 0.75] and the modified Bonded Bracket index [0.74]. The modified Turesky index produced the poorest agreement among the assessors [0.66].

The result of the study by Carter et al.^42^ confirmed the analysis of Bland–Altman diagrams in this study. The TQH index, as a modification of the international standard plaque index, was poorly quantifiable due to the structure of the scoring grades. The plaque values of the TQH index were generally too high compared to the PPI. This mainly concerned low plaque accumulations. The differences between the PPI and the TQH index were on average smaller than the differences between the PPI and the Attin index, because increased plaque accumulations were assessed more precisely with the TQH index. The Attin index produced the worst results for quantitative assessment and thus had the largest differences of the three conventional plaque indices compared with the PPI. The mBB index was the most likely to be quantitatively assessable and thus achieved the best agreement with the PPI. In general, all LoA were outside the clinical tolerance range. Consequently, none of the plaque indices studied were congruent. The TQH and mBB indices achieved the best agreement of all comparisons due to a smaller systematic error and the lowest dispersion because of the similarity of the assessment levels.

Orthodontic experience did not have a significant effect on judgment agreement. The raters with little orthodontic experience achieved the most agreement with the Attin index, and the raters with much orthodontic experience with the Attin and modified Bonded Bracket index. No difference was observed between the three conventional plaque indices among the assessors with moderate orthodontic experience. The results of our study imply that training and calibration of multiple raters are of exceptional importance for scientific studies. Therefore, it is important to ensure that raters are trained and calibrated before starting a planned study.

When examining the agreement of the plaque indices in relation to the classification of the subjects into the same oral hygiene category, similar results were obtained as in the analysis of the Bland–Altman plots. The present study showed that subjects were classified into the same oral hygiene category with two different plaque indices showing insufficient agreement. Oral hygiene categories provide an indication of a patient’s oral hygiene status and enables dental practitioners to determine an appropriate prophylaxis program. In daily clinical practice, the classification of patients into a prophylaxis program to improve or maintain oral hygiene is more important than the determination of the exact plaque score. However, for everyday clinical practice, the Attin index is recommended in the present work because the plaque index achieved good interrater reliability and was quick and easy to collect. The Attin index was developed for orthodontic patients with MBA, thus taking into account the predilection sites for plaque formation specifically in these patients. Thus, in practice, the Attin index represents a suitable method for assessing plaque accumulation and, in combination with the classification of patients into a prophylaxis program, contributes to the prevention of demineralization and gingivitis. Consequently, the various plaque indices are not interchangeable in everyday clinical practice.

In the present work, it was shown that the modified Turesky index, a dental plaque index that is considered the international standard plaque index, is less suitable for patients with MBA. Additionally, the modified Bonded Bracket index is recommended because this plaque index achieved good agreement among the assessors. The modified bonded bracket index was most likely to be quantitatively assessable and thus deviated the least from the objectively assessable plaque index percentage of all conventional plaque indices.

The following should be considered in the statistical analysis. In this study, the plaque values of the ordinally scaled plaque indices (TQH, Attin, mBB indices) were converted to metrically scaled plaque values as described in the literature and thus quantitatively assessed^24,47^. Conversion of plaque values into percentages allowed comparison of all four plaque indices despite original differences in scaling, scoring degrees, and division of tooth areas. The disadvantages of averaging and mathematical conversion were the partial loss of clinical significance of the individual plaque values. For future studies, the use of Bland–Altman plots for the comparison of plaque values of different plaque indices is not recommended, as it is a prerequisite of this statistical analysis that measurements are taken in the same units.

## Conclusion

In this study, the following three limitations were found in the application of conventional plaque indices: (1) Different degrees of evaluation and different divisions of the tooth surfaces made it difficult to compare the plaque indices with each other. (2) To standardize plaque indices, ordinal conventional plaque indices were assessed quantitatively. This led to a loss of clinical information concerning the plaque values and consequently to an inadequate interpretation of the results. (3) The subjectivity of the conventional plaque indices posed another difficulty due to the large number of raters. Our study found that plaque accumulation was rated differently with the plaque indices and thus the plaque indices were not interchangeable. Based on the results of this study, we recommend the use of the DPIA method for plaque determination for scientific studies. As an objective, quantitative, and sensitive method, DPIA allows for accurate determination of plaque accumulation independent of the assessments of individual raters. The simple statistical evaluation is a major advantage compared to conventional plaque indices. The DPIA method thus allows for an easy comparison of results from different studies and consequently leads to an improved homogeneity among studies.

## Data Availability

The data that support the findings of this study are available from PD Dr. Christina Erbe, erbe@uni-mainz.de but restrictions apply to the availability of these data, which were used under license for the current study, and so are not publicly available. Data are however available from the authors upon reasonable request and with permission of Procter & Gamble Company, Mason, Ohio.

## Abbreviations

CI: Confidence interval
DPIA: Digital plaque imaging analysis
LoA: Limits of agreement
LLoA: Lower limit of agreement
MB: Multibracket appliance
mBB Index: Modified Bonded Bracket Index
TQH Index: Modified Turesky Index
PPI: Percentage Plaque Index
ULoA: Upper limit of agreement
